# Best videos of the year for 2019

**DOI:** 10.1590/S1677-5538.IBJU.2020.01.02

**Published:** 2020-01-13

**Affiliations:** 1 Department of GU Oncology Moffitt Cancer Center, FL, USA; 2 Department of Tumor Biology Moffitt Cancer Center, FL, USA

This year has been another exceptional year for the International Brazilian Journal of Urology with a significant increase of quality video submissions. I am truthfully amazed by the innovative and beautifully depicted videos sent to us from across the world. It is through this commitment to continually refining our surgical approaches and techniques that we will set new benchmarks of excellence in our surgical specialty. In making this selection for best videos of the year, many criteria are taken into account including originality, quality of video depiction and narration, and novelty in re-defining surgical standards. In this regard, I am pleased to announce the top 3 videos of the year. The 1st Prize is awarded to the video by Garisto et al. (1) from the department of urology, Cleveland Clinic (Int Braz J Urol. 2019 Mar 22;45. [Epub ahead of print]) entitled “Robot-assisted repair for ureteroileal anastomosis stricture after cystectomy: technical points” (**see video**). Management of such strictures post-cystectomy can be quite challenging and this highly skilled team from the Cleveland Clinic led by Dr. Kaouk illustrate how it can be approached using a minimally invasive approach in a systematic and methodical manner allowing its reproducibility and adoption by others across the world. The 2nd Prize is awarded to the video by Velilla et al. (2) from the departments of urology, general surgery, and radiology at the Hospital Universitario Marques de Valdecilla in Satander, Spain (Int Braz J Urol. 2019 Mar-Apr;45(2):411). This video entitled “Robotic surgery in the management of complex pelvic endometriosis” (**see video**) highlights how minimally invasive robotic surgery can be offered to patients suffering from multi-organ site endometriosis through the coordination and team work of multi-disciplinary surgical teams. The authors nicely highlight the key steps in conducting such complex procedures offering critical details to conducting such procedures at other international centers of excellence. It is of note that embarking on such multi-organ and specialty procedures requires methodical review of pre-operative imaging and defining the affected sites of disease which will help determine the location/number of port placements, the enumerated steps of the procedure, and which surgical teams should participate and in what specific order they should proceed. This truthfully reminds me of a symphonic orchestra working in a harmonious and coordinated manner. Last but certainly not least, the 3rd prize for best video of the year is awarded to the video by Sonawane et al. (3) from the department of urology at the Muljibhai Patel Urological Hospital in Gujarat, India (Int Braz J Urol. 2019 Jan-Feb;45(1):193). This entitled “Vascular injuries during laparoscopic donor nephrectomy and proposed risk reduction strategies” (**see video**). There are many qualities related to this work including the meticulous review by the authors of their completed cases ultimately providing insight to improving patient outcomes through their only 5 cases (0.6%) of vascular injuries among their large cohort (N=858) of completed laparoscopic donor nephrectomies between 2011 and 2016. This video very elegantly depicts what can be done to avoid such injuries and if they in fact infrequently occur, how can they be managed to minimize their meaningful sequelae. There are important lessons depicted within this video that broadly apply to many facets of other laparoscopic and open surgical procedures within and outside of urology ie meticulous review of pre-operative imaging and managing such vascular complications through effective and clear communication while maintaining a calm demeanor.

In my concluding remarks, I would like to thank and congratulate our outgoing editor-in-chief Dr Sidney Glina on his exceptional leadership and vision for our journal over his very successful term. Similarly, I would like to congratulate Dr. Luciano A. Favorito in being elected as the new editor-in-chief for our journal and very much look forward to working as part of your executive editorial team.

Warmest regards and best wishes to all our readers and contributors for the holidays and the New Year. Very much looking forward to receiving your quality submissions in 2020.
